# Effect of Service Temperature on Mechanical Properties of Adhesive Joints after Hygrothermal Aging

**DOI:** 10.3390/polym13213741

**Published:** 2021-10-29

**Authors:** Wei Tan, Jingxin Na, Zhaofeng Zhou

**Affiliations:** 1 School of Engineering, Zhejiang University City College, Hangzhou 310015, China; tanweidd18@163.com; 2 State Key Laboratory of Automotive Simulation and Control, Jilin University, Changchun 130031, China; najx@jlu.edu.cn

**Keywords:** polyurethane adhesive, service temperature, hygrothermal aging, failure mechanism, failure criterion

## Abstract

Polyurethane adhesive and aluminum alloy were selected to make adhesive joints. Butt joints tested at different loading angles (0°, 45°, and 90°) using a modified Arcan fixture were selected to represent three stress states (normal stress, normal/shear combined stress, and shear stress, respectively). Firstly, the accelerated aging tests were carried out on the joints in a hygrothermal environment (80 °C/95% RH). The quasi-static tests were carried out at different temperatures (−40 °C, 20 °C, and 80 °C) for the joints after hygrothermal aging for different periods. The variation rules of the joints’ mechanical properties and failure modes with different aging levels were studied. The results show that the failure load of the joints was obviously affected by stress state and temperature. In the low-temperature test, the failure load of the joints decreased most obviously, and the BJ was the most sensitive to temperature, indicating that the failure load decreased more with the increase of the normal stress ratio in the joint. Through macroscopic and SEM analysis of the failure section, it was found that the hydrolysis reaction of polyurethane adhesive itself and the interface failure of the joints were the main reasons for the decrease of joint strength. The failure models were established to characterize the adhesive structure with different aging levels at service temperature.

## 1. Introduction

**Figure 1:**
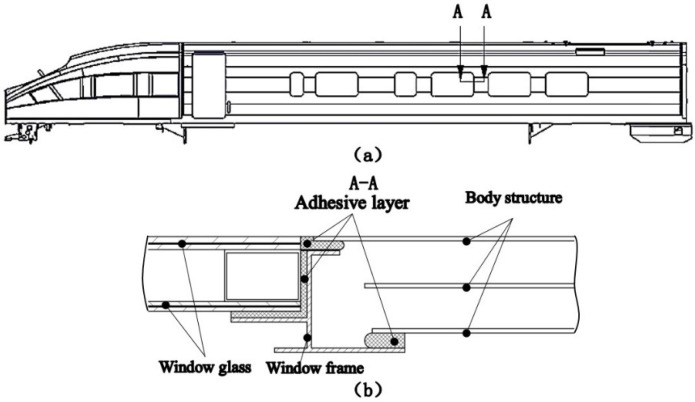
(**a**) Schematic diagram of the high-speed train, (**b**) schematic diagram of side window adhesive structure.

Adhesion technology has been widely used in the assembly process of high-speed train components. For example, the side window structure, side skirt structure, and curved head system of the CRH1–CRH13 series and CRH380 series trains are all connected to the train frame through adhesive. In Figure 1, the side window adhesive structure of the high-speed train is shown. Different heat-conduction coefficients between the window glass and car body frame lead to different deformation when the temperature changes. The adhesive layer must ensure a certain thickness and deformation ability, so for the connection between the window and car body frame, flexible elastic adhesion technology is chosen. The window glass and the car body frame are elastically bonded to form the window component unit. Elastic adhesion technology improves airtightness, reduces the penetration of water vapor, mitigates the impact and vibration of the side window when the high-speed train passes by or through a tunnel, reduces the aerodynamic noise inside the vehicle, and ensures the safety and riding comfort of the train [1].

In the service process of the high-speed trains, the service environment for the body structure is complex, and the service temperature range (−40–80 °C) has a large span. Adhesives belong to the high-molecular compounds. As a temperature-sensitive material, the temperature directly affects the mechanical properties of the material, and its strength and failure mode change with the temperature [2]. Therefore, in vehicle operation, the adhesive structure needed to provide sufficient strength must be within the service temperature range. Simultaneously, in the service process of the high-speed trains, the adhesive structure is more vulnerable to the comprehensive effect of environmental factors, such as temperature and humidity. The adhesive can age under the long-term impact of temperature and humidity, and its chemical composition can also change. The temperature and humidity in the environment are the main factors causing the aging of materials [3].

Domestic and foreign scholars have carried out the following research on the influence of temperature on the static properties of adhesive structures. The mechanical properties of adhesives changed in different temperature ranges, while the adhesive strength, strain, and fracture toughness showed temperature dependence [4,5]. The impact of temperature on the properties of adhesive structure was noticeable, especially when the temperature was close to the glass transition temperature (T_g_) of the material [6,7]. When the temperature was higher than T_g_, adhesive exhibited a high elastic state, the failure strength and elastic modulus decreased rapidly, and the elongation increased. However, when the temperature was lower than T_g_, its performance was reversed [8]. Na et al. [9,10] studied the effect of temperature on the mechanical properties of adhesive joints. They found that with the increase of temperature, the Young’s modulus and tensile strength of the joints decreased, while the tensile strain increased. The closer to T_g_, the more significant the change of mechanical properties. Silva et al. [11] tested the mechanical properties of a single lap joint at low temperature and high temperature, finding that the adhesive was brittle at low temperature and ductile at high temperature, and analyzed the effect of porosity on failure.

At the same time, domestic and foreign scholars have carried out relevant research on the influence of hygrothermal aging on adhesive structures’ strength property. It was found that the aging behavior of the adhesive system in the service process of the high-speed trains was usually the result of the combined effect of temperature and humidity. The most commonly used durability test method was to carry out accelerated aging tests on adhesive joints through temperature-humidity coupling conditions [12]. Under the coupling effect of temperature and humidity, the moisture absorption of the adhesive would cause plasticization and expansion, and the difference of thermal expansion coefficient between the adhesive and the substrate would cause thermal stress. In addition to the increase of strain, the degradation of moisture would significantly reduce the strength, stiffness, and fracture toughness of the adhesive [3], indicating that moisture reduced the strength and service life of the joints, and that the durability of the adhesive structure was affected by multiple factors’ comprehensive results [13]. In the service process of adhesive structure, especially in a hygrothermal environment, the moisture diffusion intensified the moisture absorption of the adhesive [14]. Heshmati and Viana et al. [15,16] studied the aging behavior of adhesive joints in a hygrothermal environment, quantitatively evaluated the adhesives and joints, and analyzed the environmental degradation mechanism. High-speed trains’ adhesive structure was affected not only by hygrothermal aging but also by the complex stress state in the service process. Simultaneously, the adhesive’s temperature sensitivity made the mechanical properties and failure mode of the adhesive structure change with the temperature. Therefore, the design’s strength at room temperature could not meet the adhesive structure’s requirements for the whole service temperature range. In this paper, considering the impact of aging and temperature, quasi-static tests of different aging levels of joints at different temperatures were carried out to study the influence of temperature on the mechanical properties and failure modes. The accelerated aging test of the adhesive joints was carried out in a hygrothermal environment (80 °C/95% RH) with aging periods of 0, 6, 12, 18, 24, and 30 days, respectively. Then, the quasi-static mechanical properties of the joints with different aging periods were tested at different temperatures (−40 °C, 20 °C, and 80 °C). Through the mechanical properties test under the quasi-static load of the complex stress state, the influence of the stress state on the strength of the joints was studied under the three conditions of tension, shear, and tension/shear mixed loading. The failure mode was studied by analyzing the cross-section of the joints, and SEM analyzed the microstructure and failure mechanism. Finally, the failure models of adhesive structures with different aging levels under service temperature were established. The failure criteria related to aging cycle and temperature were obtained, which could be used to check the strength under different aging levels and temperatures.

## 2. Experimental Study

### 2.1. Materials

In this paper, 6005A aluminum alloy, one of the 6000 series aluminum alloys, was selected as the bonding substrate. It mainly contains Al, Mg, and Si metal elements. It has high strength, good plasticity, and corrosion resistance, making it ideal to replace the heavier materials in high-speed trains to meet the weight reduction requirements. It is a commonly used lightweight material in high-speed trains. The primary technical parameters of 6005A aluminum alloy (data provided by the supplier) are shown in Table 1.

The structural adhesive studied is Sikaflex^®^-265, a one-component polyurethane adhesive produced by Sika Company (Baar, Switzerland). It can be self-cured by absorbing moisture in the air when exposed to atmospheric humidity, forming a permanent elastomer after curing. It has a wide range of applications, such as rail trains, buses, and trucks. The adhesive has excellent high ductility, fatigue durability, impact resistance, high toughness, and so on. It can realize the elastic connection of the bonding substrate, avoid the stress concentration of the connection structure, and improve the fatigue performance, and has the advantages of shock absorption and noise reduction. The primary technical parameters of Sikaflex^®^-265 adhesive are shown in Table 2 [17].

### 2.2. Joint Type

**Figure 2:**
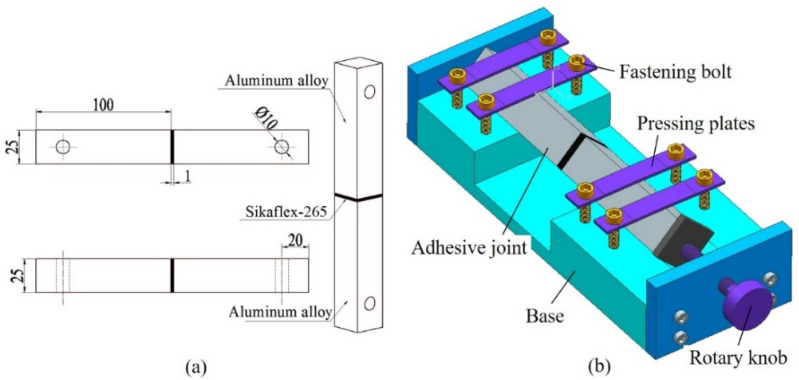
(**a**) Geometric dimension of BJ (unit: mm), (**b**) schematic diagram of bonding fixture.

To study the mechanical properties of the adhesive joints under normal stress, a butt joint is designed and manufactured. The butt joint is not in a uniaxial stress state but in a multiaxial stress state. Although the stress state near the free edge of the butt joint is a combination of shear stress and normal stress, and there is a significant stress concentration, the main stress inside the adhesive layer of the joint is normal stress [18,19]. The normal stress distribution is uniform except for the end of the adhesive layer. The assumption that normal stress is expressed by the butt joint is acceptable in engineering practice. The geometry and size of the butt joint are shown in Figure 2a. The overall size of the joint is 201 × 25 × 25 mm^3^, the bonding area is 25 × 25 mm^2^, and the adhesive layer thickness is 1 mm.

The joints were prepared in a dust-free and stable environment (temperature 25 ± 5 °C, humidity 50 ± 5%). To avoid the failure of bonding due to improper surface treatment, the surface of the bonding substrate was treated first, and the aluminum alloy was cross-polished with 80 mesh sandpaper along the diagonal direction of the bonding surface to increase the surface roughness. Sika Remover 208, Sika Aktivator, and Sika Prime-206G + P were used successively to wipe the bonded surface [20]. When all the above pretreatment processes were completed, glue was applied on the surface of the aluminum alloy substrate, and the adhesive fixture was used for bonding, as shown in Figure 2b. The distance between the upper and lower aluminum alloy substrates was controlled by rotating the handle, and a vernier caliper measured the relative size of the intermediate adhesive layer, so as to better control the thickness of the adhesive layer. The joints were cured for 4 weeks under the condition of temperature 25 ± 5 °C and humidity 50 ± 5%, and then the residual glue was cleaned for the next test.

### 2.3. Joint Stress Distribution and Arcan Fixture Test

**Figure 3:**
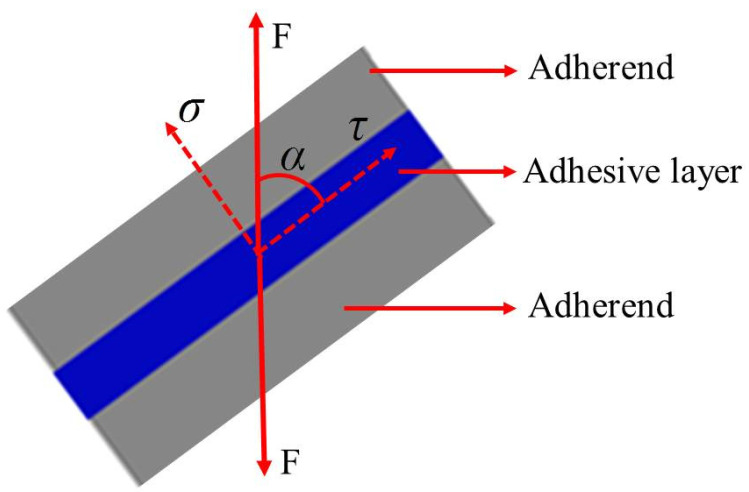
Stress vector in the adhesive layer of the adhesive joint.

The adhesive structure in engineering is often subjected to tension, shear, bending, torsion, or composite load, which means the adhesive layer is always in a normal/shear stress state. Because the combined loading of tensile and shear loads is a simple method to determine the relationship between material strength and constitutive stress, the quasi-static loading test of mixed loads has been widely used. The combined loading of tensile and shear loads will produce different stress states, from pure normal stress to pure shear stress. According to the angle of each loading condition, the failure stress vector is divided into normal stress and shear stress, as shown in Figure 3. The normal stress and shear stress components are given by Equations (1) and (2).

(1) σ=F⋅sin α/S

(2) τ=F⋅cos α/S

where σ is the normal stress, MPa; τ is the shear stress, MPa; *F* is the failure load, *N*; *S* is the bonding area, mm^2^; and *α* is the loading angle.

In this study, butt joints tested at different loading angles (0°, 45°, and 90°) using a modified Arcan assembly were selected to represent three stress states (normal stress, normal/shear combined stress, and shear stress, respectively). For convenience of description, they are denoted by BJ, 45° SJ, and TASJ, respectively. The 45° SJ (*α* = 45°) is subjected to a combination of shear stress and normal stress, and the ratio of the normal stress to the shear stress component of the joint is 1, while the *α* of TASJ and BJ is 0° and 90°, respectively. The ratio of normal stress σ to shear stress τ for TASJ, 45° SJ, and BJ is 0, 1, and +∞, respectively. Hence, BJ has the highest proportion of normal stress in the adhesive layer, followed in decreasing order by 45° SJ and TASJ.

**Figure 4:**
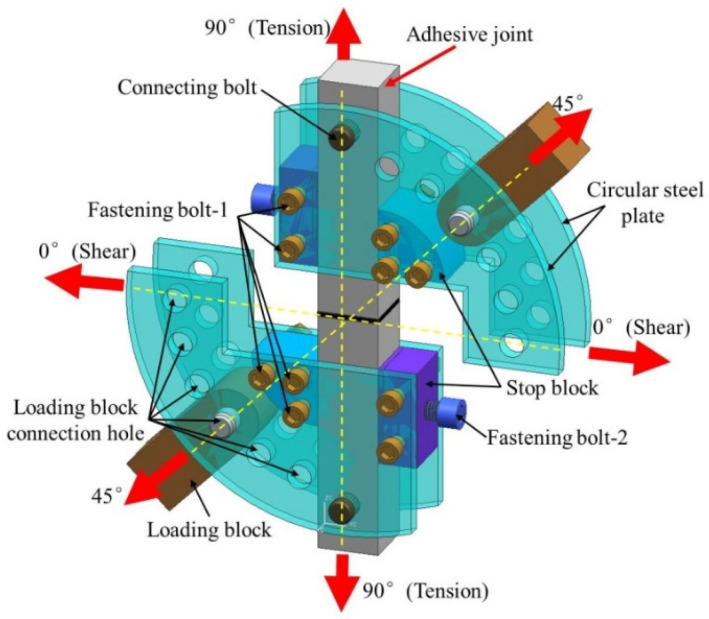
Schematic diagram of Arcan fixture.

To study the adhesive joints under different stress states, an improved Arcan fixture (as shown in Figure 4) was developed and manufactured, which could realize the combined loading of tensile and shear stress [21]. The fixture was mainly composed of semicircular steel plates, loading blocks, positioning blocks, limiting blocks, connecting bolts, positioning bolts, and fastening bolts. The semicircular steel plate consisted of four independent steel plates with multiple holes, the thickness of which was 6 mm. The holes on the steel plates were respectively used for connection, limit, and positioning, so that the loading direction was related to the joint movement. Positioning blocks, limiting blocks, fastening bolts, and positioning bolts were set on both sides of the adhesive joint to fix and determine the joint. Fastening bolts connected the positioning blocks, the limiting blocks, and the semicircular steel plates. Two connecting bolts were used to connect the adhesive joint to ensure uniform load transfer. The specific stress state could be obtained at the adhesive joint by loading the circular steel plate with different angles by the loading blocks.

### 2.4. Accelerated Aging and Quasi-Static Test

Artificial accelerated aging was used to accelerate the degradation of the adhesive. The change law of its mechanical properties and failure mechanism was analyzed to simulate the harsh environmental conditions encountered in the service process of high-speed trains [12]. According to the extreme environmental conditions of the high-speed train service environment, referring to the standard “Use of adhesive in the manufacture of rail vehicles and parts of rail vehicles” (DIN6701-2:2015-12), the accelerated aging test was carried out in a hygrothermal environment (80 °C/95% RH) to analyze the influence of aging on the joint’s mechanical properties. The aging cycles were 0, 6, 12, 18, 24, and 30 days, respectively. It was found that at 80 °C/95% RH, the mass of adhesive increased and reached moisture equilibrium after 72 h [17]. The accelerated aging test was carried out in a WSHW-080BF hygrothermal environment chamber (WEISS Experimental Equipment Inc., Jiaxing, Zhejiang, China).

**Figure 5:**
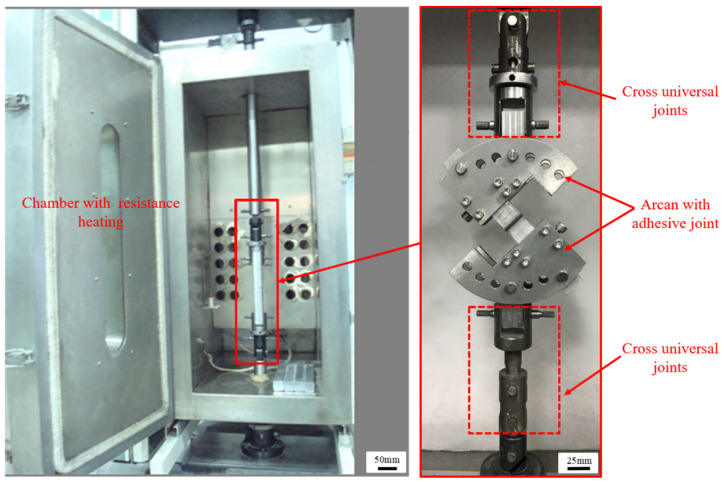
Quasi-static mechanical properties test at different temperatures.

The previous test [17] found that hygrothermal environment (80 °C/95% RH) aging had obvious effect on the mechanical properties of the joints. In order to investigate the impact of service temperature on the adhesive joints’ mechanical properties after hygrothermal aging, the quasi-static tests of the joints after hygrothermal aging were carried out at different temperatures (−40 °C, 20 °C, and 80 °C). Firstly, the adhesive joints were subjected to varying cycles of hygrothermal aging tests. Secondly, the aged adhesive joints were left at room temperature for 24 h. Then, the aged adhesive joints were placed in an environment chamber, the specific temperature was set, and the joints were allowed to stand for 2 h, waiting for the internal temperature of the joints to be fully mixed and uniform. Finally, the quasi-static mechanical tests were carried out by using an electronic universal testing machine (WDW series, Kexin Inc., Changchun, Jilin, China, as shown in Figure 5) with the high- and low-temperature environment chamber. To eliminate the non-axial force, both ends of the joints were connected to the testing machine through a universal joint-like structure, and the joints were tested at a constant rate of 5 mm/min until fracture. The load-displacement curve of each joint was obtained. The failure load and failure strength of the joints were compared and analyzed. Each test condition was repeated four times. The macroscopic and microscopic morphology of the failure sections were discussed, and the failure mechanism was analyzed.

## 3. Results and Analysis

### 3.1. Failure Load Analysis

**Figure 6:**
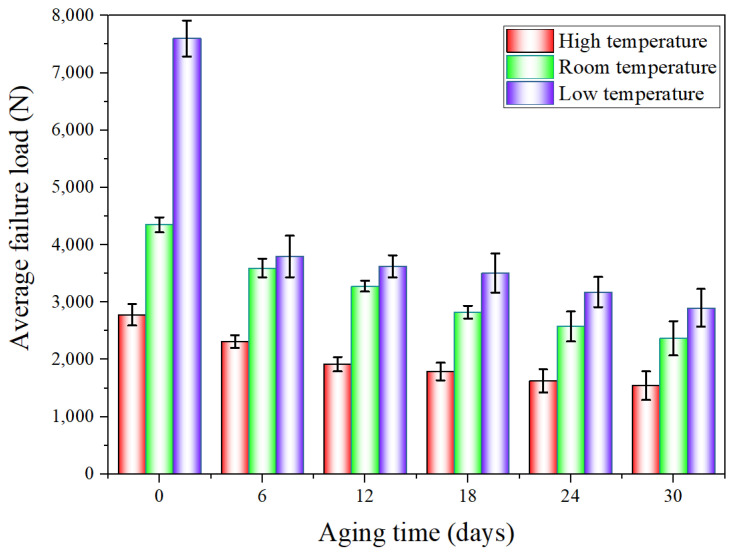
The average failure load of BJs after hygrothermal aging at different temperatures.

**Figure 7:**
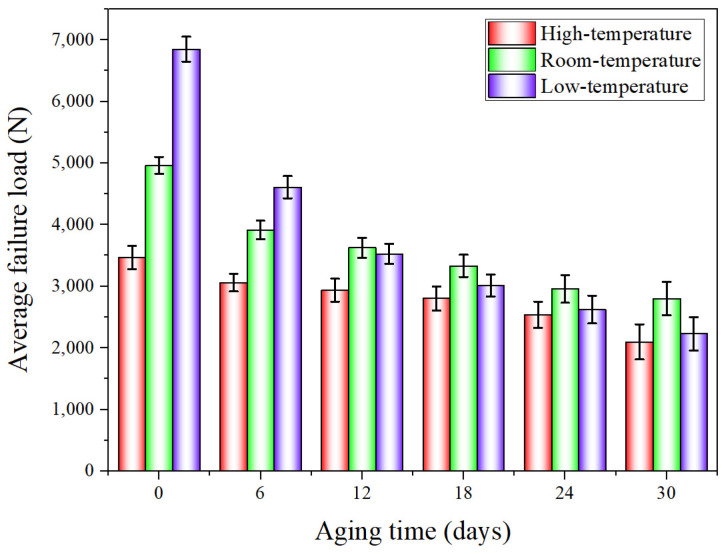
The average failure load of 45° SJs after hygrothermal aging at different temperatures.

**Figure 8:**
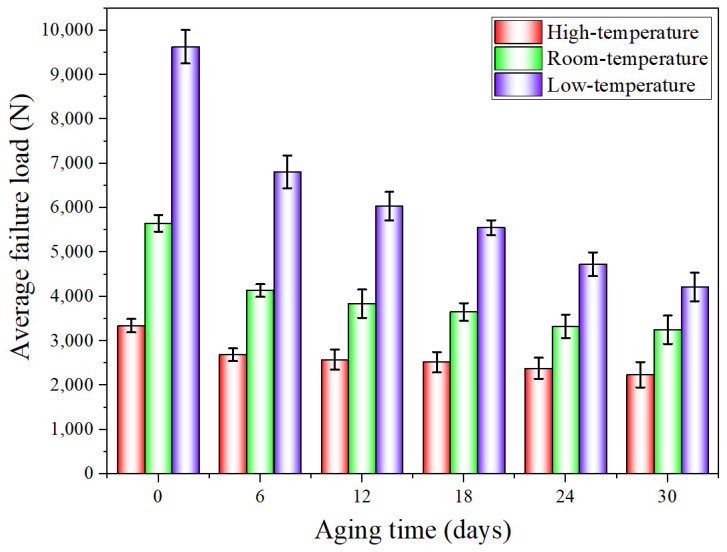
The average failure load of TASJs after hygrothermal aging at different temperatures.

When the quasi-static mechanical properties of the adhesive joints after hygrothermal aging were tested at three temperatures (−40 °C, 20 °C, and 80 °C), the obtained mechanical properties data were statistically processed to analyze the variation law of the average failure loads of BJs, 45° SJs, and TASJs, as shown in Figure 6, Figure 7 and Figure 8. It is found that the average failure loads of the joints decrease gradually with the increase of aging time, and there are apparent differences in the variation range of failure load under different temperatures and different stress states.

The average failure loads of BJs are shown in Figure 6. At high temperature, compared with unaged, the failure load decreases by 16.9%, 31.1%, 35.7%, 41.6%, and 44.4% at 6, 12, 18, 24, and 30 days of hygrothermal aging. At room temperature, compared with unaged, the failure load decreases by 17.4%, 24.7%, 35.1%, 40.8%, and 45.6% at 6, 12, 18, 24, and 30 days of hygrothermal aging. At low temperature, compared with unaged, the failure loads at 6, 12, 18, 24, and 30 days of hygrothermal aging decreases by 52.7%, 56.9%, 62.8%, 66.1%, and 68.8%, respectively.

It is found that the failure loads of the joints decrease obviously at the initial stage, and the decline rate gradually decreases with the increasing of the aging time. After aging for 6 days, the failure loads obviously decrease; this is because the polymer chain breaking for the longest duration, the fastest breaking speed, and the fastest decreasing cross-linking density are all seen at the early aging stage [22], leading to a significant reduction of the average failure loads of the joints. The results show that the interface failure of the joints at low temperature obviously occurs after 6 days (see the discussion in Section 3.3), which reflects the apparent change of failure load. The failure load at low temperature decreases to the largest extent, while the decline at high temperature is the least, which also shows that the joints’ mechanical properties at low temperature decrease most obviously after hygrothermal aging. In addition, the data dispersion increases at low temperature, which indicates that the data consistency is poor at low temperature.

The average failure loads of the 45° SJs are shown in Figure 7. At high temperature, compared with unaged, the failure load decreases by 11.7%, 15.2%, 19.1%, 26.8%, and 39.5% at 6, 12, 18, 24, and 30 days of hygrothermal aging. At room temperature, compared with unaged, the failure load decreases by 21.2%, 26.9%, 32.9%, 40.4%, and 43.6% at 6, 12, 18, 24, and 30 days of hygrothermal aging. At low temperature, compared with unaged, the failure loads at 6, 12, 18, 24, and 30 days of hygrothermal aging decreases by 32.7%, 48.5%, 56.0%, 61.7%, and 67.5%, respectively. The failure load decreases obviously after aging for 6 days, which is also due to the apparent interface failure of the failure section at low temperature after aging for 6 days, and the decline rate gradually decreases with the increase of aging time.

The average failure loads of TASJs are shown in Figure 8. At high temperature, compared with unaged, the failure load decreases by 19.5%, 22.9%, 24.6%, 28.7%, and 33.0% at 6, 12, 18, 24, and 30 days of hygrothermal aging. At room temperature, compared with unaged, the failure load decreases by 26.9%, 32.1%, 35.4%, 41.2%, and 42.5% at 6, 12, 18, 24, and 30 days of hygrothermal aging. At low temperature, compared with unaged, the failure loads at 6, 12, 18, 24, and 30 days of hygrothermal aging decrease by 29.3%, 37.3%, 42.3%, 50.9%, and 56.2%, respectively. With the increase of aging time, the decline rate of failure load decreases gradually. After hygrothermal aging, the failure load at low temperature reduces the most. In contrast, at high temperature, it decreases the least, which indicates that the mechanical properties at low-temperature reduce most obviously after hygrothermal aging.

The above research found that the stress state and temperature have significant effects on the failure load of the adhesive joints after hygrothermal aging. With the increase of aging time, the decline rate of the failure load decreases gradually. Compared with the failure load measured at three temperatures after hygrothermal aging, the decrease of BJ is the most obvious, while the decline of TASJ is the smallest, which indicates that BJ is the most sensitive to temperature. With the increase of the proportion of normal stress in the joint, the decrease of failure load increases gradually. Simultaneously, compared with the failure load of three types of joints tested after hygrothermal aging, the failure loads decrease most obviously at low temperature, while the decline is the least at high temperature, which is closely related to the failure modes of joints tested at different temperatures. The following Section 3.3 will focus on the discussion and analysis.

### 3.2. Failure Strength Degradation Model

Considering the influence of service temperature on the adhesive structure’s mechanical properties after hygrothermal aging, the change law of the adhesive joints’ failure strength was analyzed. Furthermore, the failure strength prediction model of the aging level of the joints with time under different temperatures was established.

**Figure 9:**
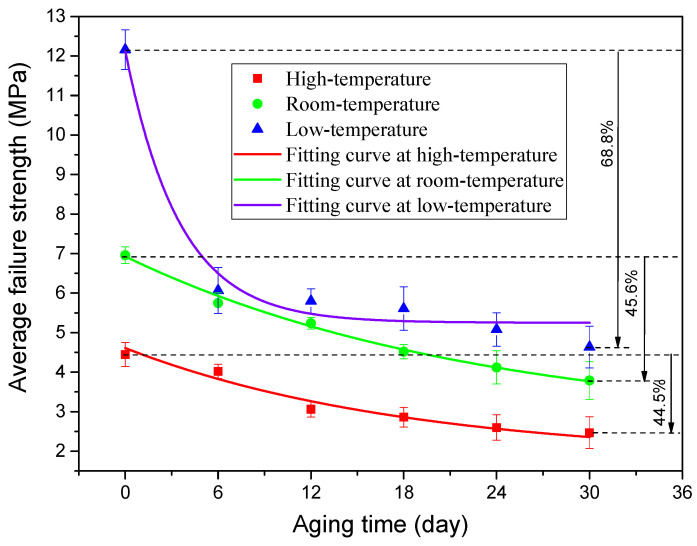
Fitting curves of average failure strength of BJs.

**Figure 10:**
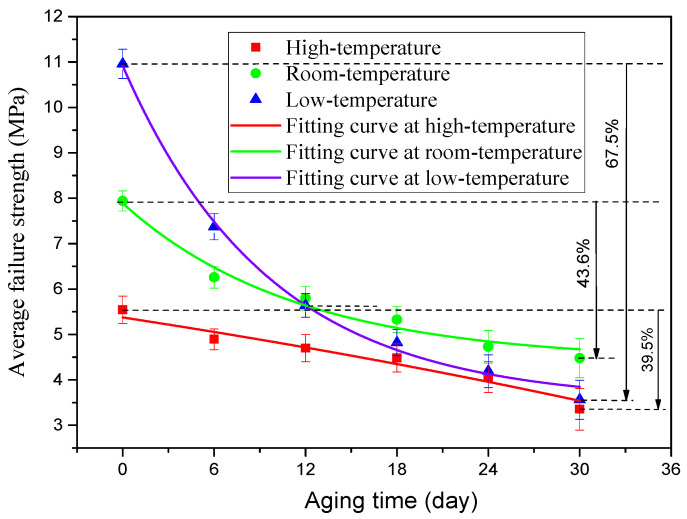
Fitting curves of average failure strength of 45° SJ.

**Figure 11:**
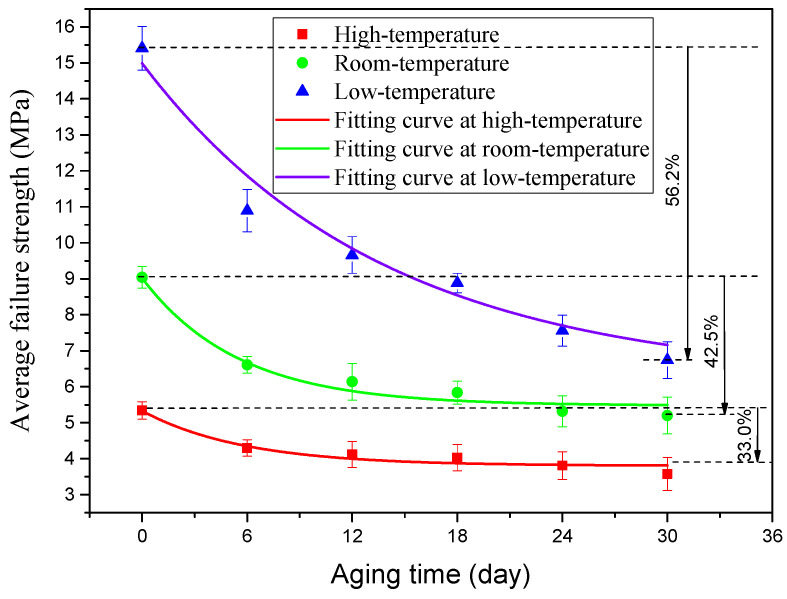
Fitting curves of average failure strength of TASJ.

To obtain the change rule of the joint average failure strength with aging time, according to the changing trend of the joint failure strength, the exponential function was used to fit the data, and the fitting curves of three types of adhesive joints were obtained, as shown in Figure 9, Figure 10 and Figure 11. The failure strength decreases with aging time. With the increase of test temperature, the failure strength decreases slowly. With the decrease of the proportion of normal stress in the joint, the decline of failure strength decreases gradually. The function expressions of the fitting curves are shown in Table 3. It is found that the fitting accuracy *R*^2^ is above 0.90, which indicates that the exponential function can obtain very satisfactory fitting accuracy. From the overall trend, the fitting impact is better at room temperature and low temperature, which indicates that the dispersion of joint failure strength is small. At the same time, it is relatively poor at high temperature.

### 3.3. Failure Section Analysis

**Figure 12:**
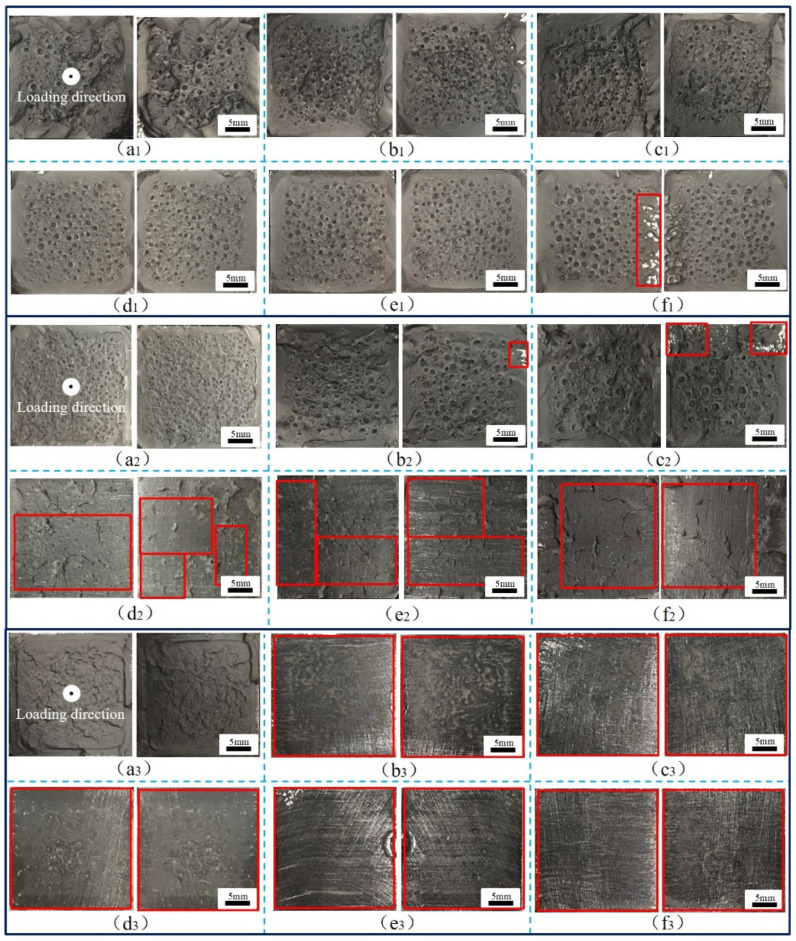
BJs, high temperature: (**a_1_**) 0, (**b_1_**) 6, (**c_1_**) 12, (**d_1_**) 18, (**e_1_**) 24, (**f_1_**) 30 days; room-temperature: (**a_2_**) 0, (**b_2_**) 6, (**c_2_**) 12, (**d_2_**) 18, (**e_2_**) 24, (**f_2_**) 30 days; low temperature: (**a_3_**) 0, (**b_3_**) 6, (**c_3_**) 12, (**d_3_**) 18, (**e_3_**) 24, (**f_3_**) 30 days.

**Figure 13:**
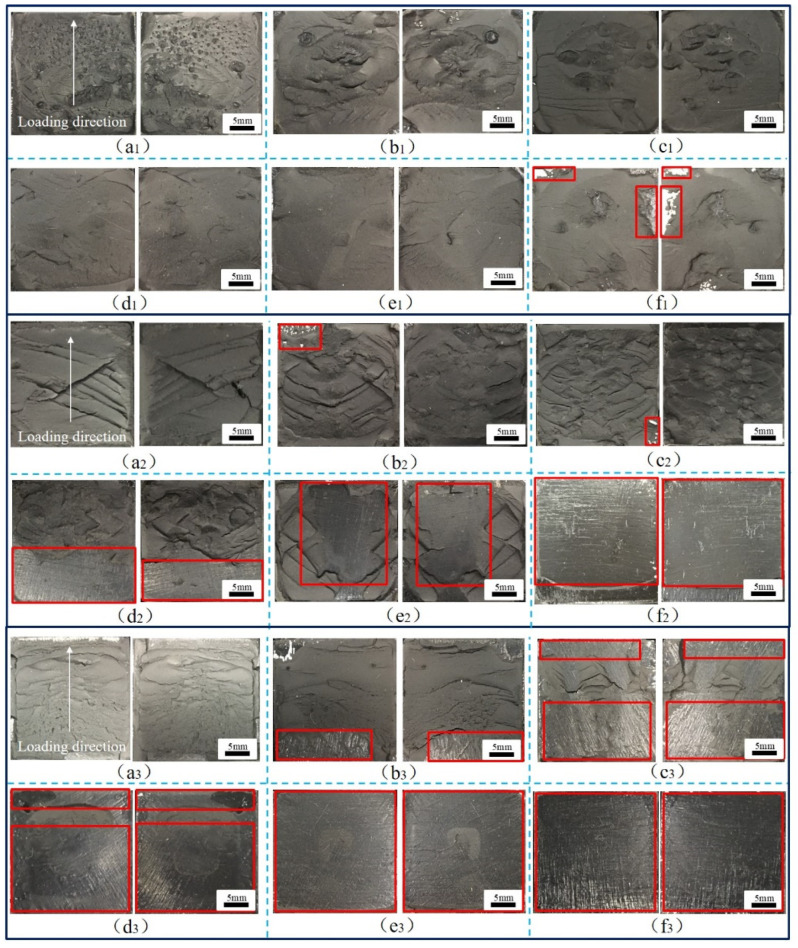
Images of 45° SJs, high temperature: (**a_1_**) 0, (**b_1_**) 6, (**c_1_**) 12, (**d_1_**) 18, (**e_1_**) 24, (**f_1_**) 30 days; room temperature: (**a_2_**) 0, (**b_2_**) 6, (**c_2_**) 12, (**d_2_**) 18, (**e_2_**) 24, (**f_2_**) 30 days; low temperature: (**a_3_**) 0, (**b_3_**) 6, (**c_3_**) 12, (**d_3_**) 18, (**e_3_**) 24, (**f_3_**) 30 days.

**Figure 14:**
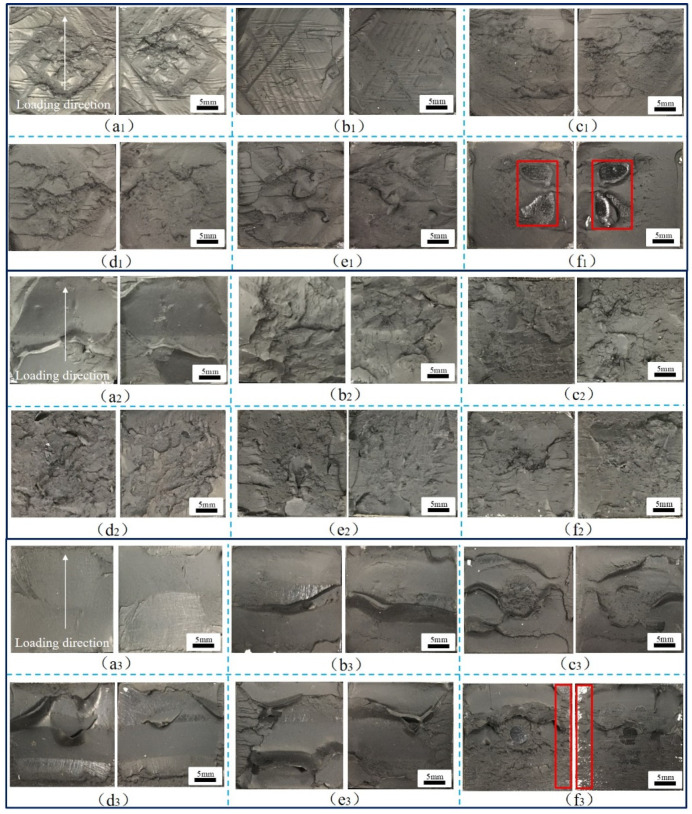
TASJs, high temperature: (**a_1_**) 0, (**b_1_**) 6, (**c_1_**) 12, (**d_1_**) 18, (**e_1_**) 24, (**f_1_**) 30 days; room temperature: (**a_2_**) 0, (**b_2_**) 6, (**c_2_**) 12, (**d_2_**) 18, (**e_2_**) 24, (**f_2_**) 30 days; low temperature: (**a_3_**) 0, (**b_3_**) 6, (**c_3_**) 12, (**d_3_**) 18, (**e_3_**) 24, (**f_3_**) 30 days.

During the operation of a high-speed railway train, the adhesive structure of the train body is in a complex stress state, and the failure modes of the adhesive system are complex, including cohesive failure, interface failure, and mixed failure. In addition, the aging of adhesive will reduce the strength of the adhesive structure and directly affect the failure mode under a complex stress state. Therefore, it is necessary to establish accurate failure models of adhesive structures with aging cycles at different temperatures, analyze the crack evolution behavior of adhesive joints under aging and temperature coupling effects, and reveal the mechanism of action and failure. By analyzing the failure sections of the adhesive joints, the typical failure sections of BJs, 45° SJs, and TASJs under different temperature conditions after hygrothermal aging are obtained, as shown in Figure 12, Figure 13 and Figure 14 (the red box indicates the interface failure area).

The BJs with different aging cycles are tested at different temperatures, and the failure sections are shown in Figure 12. It can be seen that the apparent feature of BJ is that there are many holes on the surface of the failure section, which is caused by cavitation [23], which is considered to be the primary mechanism that causes the failure of the adhesive layer of the BJ [24]. In the tensile process, the formation of holes was more likely to cause cracks in the adhesive layer, accelerate the diffusion of water, and cause fracture, thus reducing the joint strength more quickly. At low temperature, close to T_g_, the adhesive showed more toughness. The shrinkage of the hole at low temperature leading to the cavitation on the failure section was not obvious, and there was an obvious crack phenomenon. With the increase of temperature, the temperature was much higher than T_g_ of the adhesive, and the adhesive was more elastic. The holes expanded at high temperature, and the number of holes in the failure section decreased, but the volume increased. It was found that interface failure was more likely to occur in the edge area of the failure section, because the edge was more prone to cracking under the action of stress, which accelerated the diffusion of moisture from the boundary of the adhesive layer to the interior, causing the expansion of the adhesive layer, and weakening the bonding force between the adhesive and the bonding substrate [25].

Simultaneously, it was found that cohesive failure mainly occurred in the failure section at high temperature, and there was a small area of interface failure at 30 days of aging. At room temperature, with the increase of aging time, the failure section began to change from cohesive failure to mixed failure, and the interface failure was severe at 18 days. However, at low temperature, the failure section of the joint changed obviously. Only after 6 days of aging did the failure section of the joint turn into interface failure, which provided a reasonable explanation for the apparent decrease of failure strength at low temperature after aging for 6 days.

It was further found that at the same temperature, the proportion of interface failure in the failure section increased with the increase of aging time. This is mainly because water molecules could easily penetrate the interface between the adhesive layer and the bonding substrate under a hygrothermal environment, leading to the volume expansion of the interface adhesive layer, resulting in the concentration of internal stress in the joints. Under the action of stress, cracks appeared easily, reducing the interfacial force between the adhesive and the bonding substrate [26]. When tested at low temperature, the temperature was close to the T_g_ of the adhesive, and the strength of the adhesive was greater than the interfacial force between the adhesive and the bonding substrate, resulting in the interface failure of the joint. At high temperature, the temperature was much higher than the T_g_ of the adhesive; the adhesive strength was significantly reduced, and was less than the interfacial force, which quickly led to the cohesive failure of the joint.

The different aging cycles of the 45° SJs were tested at different temperatures, and the failure sections were obtained, as shown in Figure 13. It can be seen that there are no bubbles on the fracture surface of the joints because the joints are in the composite state of shear and normal stress, which is not conducive to cavitation under the action of shear stress. In the high-temperature test, the failure sections mainly occurred by cohesive failure. With the increase of aging time, the failure sections gradually tended to be smooth, and there were small areas of interface failure at 30 days of aging. With the aging time increasing, the failure sections began to change from cohesive failure to mixed failure at room temperature. Compared with room temperature, the failure sections were more prone to interface failure with the increase of aging time at low temperature. The failure sections were completely interface failure after 24 days of aging. It was found that at the same temperature, the proportion of interface failure increased with the increase of aging time. With the decrease of temperature, the failure sections of the joints changed obviously, and interface failure was more likely to occur.

The TASJs with different aging cycles are tested at different temperatures, and the failure sections are shown in Figure 14. It can be seen from the figure that the failure section changes obviously at different temperatures, and the failure mode of the joints is a mainly cohesive failure. The failure section of the unaged joints at −40 °C was smooth and flat, and there was no noticeable bulge. With the increase of temperature, many cracks appeared based on folds on the failure section at 80 °C, with a shallow crack depth but more intensive cracks.

The reason is that the adhesive temperature is close to the T_g_ at low temperature, showing the morphology of ductile fracture, which leads to the smoother failure section. As the temperature increased, the viscoelastic characteristics of the adhesive became more apparent, which led to an increase in the number of folds and cracks in the failure section. When the adhesive joints after hygrothermal aging were tested at room temperature and low temperature, the failure section was smooth, and it was smooth at the initial stage of aging. Still, with the increase of aging time, the section became rougher, and noticeable folds, cracks, and protrusion appeared. However, when tested at high temperature, the failure section had no obvious change rule, although a certain degree of interface failure occurred at 30 days.

The above analysis shows that the hygrothermal environment is conducive to the diffusion of moisture in the adhesive layer. The morphology of the failure section changes significantly, especially in the edge area. At the same time, during the service of the adhesive structure, micro-cracks and holes appear in the adhesive layer and the adhesive substrate. With the evolution and expansion of micro cracks and holes, macro cracks are formed until fracture failure. More importantly, with the decrease of test temperature, the failure section of the joints changes significantly, and interface failure is more likely to occur, which indicates that the failure mechanism of the joint changes when the test is carried out at different temperatures.

### 3.4. SEM Analysis

**Figure 15:**
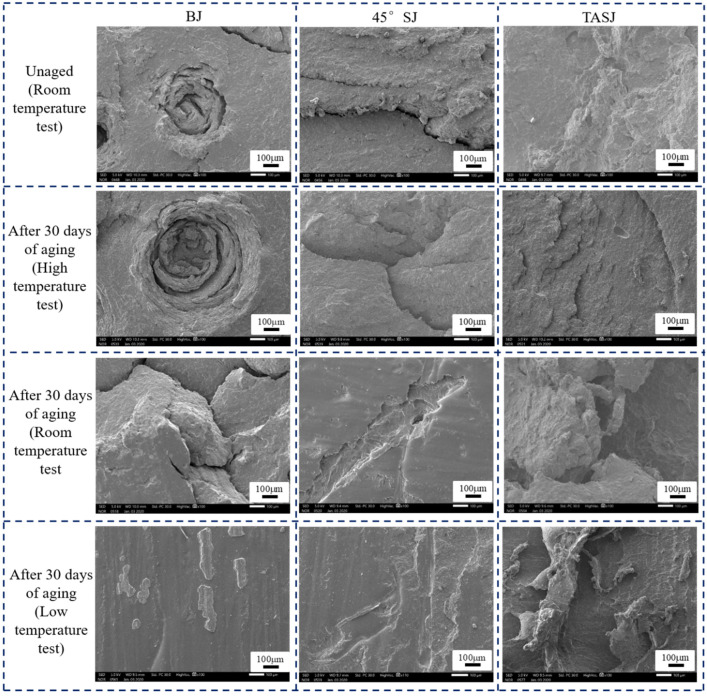
Effect of service temperature on failure section of aged BJs, 45° SJs, and TASJs by SEM (100×).

Through SEM, we studied the fracture morphology of the aged joints at different temperatures. Figure 15 shows SEM (100×) micrographs of the fracture surfaces of BJs, 45° SJs, and TASJs without aging and after 30 days of hygrothermal aging.

It is found that the failure section of the BJ without aging is smooth, with small holes and fewer cracks. After 30 days of hydrothermal aging, the size of holes in the failure section of the joint increases at high temperature, and the crack phenomenon is apparent. At room temperature, the holes in the failure section disappear, and apparent cracks appear. The failure section has complete interface failure at low temperature, and the micro section is smooth and flat. The failure section of the 45° SJs without aging has no obvious crack, and the surface is smooth. After 30 days of hygrothermal aging, although cracks exist in the failure section of the joint tested at high temperature, the micro section is relatively flat on the whole. The failure section changes significantly at room temperature, resulting in interface failure. The failure section at low temperature is also an obvious interface failure. The results show that there is only a slight bulge and no micro crack in the failure section of the TASJ before aging. With the decrease of test temperature, the bulging phenomenon in the micromorphology of the failure section becomes more and more obvious.

The failure section’s micromorphology verified that the adhesive joint’s failure mechanism after hygrothermal aging changes obviously when the quasi-static test is carried out at different temperatures. It is found that the water molecules can easily penetrate into the interface between the adhesive layer and the bonding substrate under a hygrothermal environment, and the water molecules infiltrating into molecules leads to a chemical degradation reaction with the adhesive. The interface failure of the joint and the hydrolysis reaction of the polyurethane adhesive itself are the main reasons for the strength reduction of the joint.

## 4. Failure Criterion

### 4.1. Failure Criterion Theory

In the service process of the high-speed train’s adhesive structure, the stress state is complex, and the service temperature varies widely. The temperature sensitivity of the adhesive makes the mechanical properties and failure mode of the adhesive structure change with the temperature. The design strength at room temperature can’t meet the requirements of the whole service temperature range of the train adhesive structure. In this section, the influences of hygrothermal aging and temperature are considered. The failure prediction model of the adhesive structure in the service temperature range is established, which provides the basis for the mechanical properties of the adhesive system in the whole service temperature range.

Because the adhesive structure layer is thin, its stress forms mainly include normal stress perpendicular to the bonding interface and shear stress parallel to the bonding interface. Considering the failure prediction under mixed mode, the quadratic stress criterion is selected. The quadratic stress criterion based on normal stress and shear stress is widely used in the failure prediction of adhesive structures [27]. The failure criterion can predict the failure of tensile/shear combined stress state. By fitting the joints’ normal stress and shear stress under three stress states, the quadratic stress failure criterion of the joints is established [28]. The expression of the stress failure criterion is as follows:

(3) $${\left(\frac{\tau }{S}\right)}^{q}+{\left(\frac{\sigma }{N}\right)}^{q}=1$$

where, σ and τ represent normal stress and shear stress of adhesive layer, respectively. *N* and *S* represent failure strength of mode I (tensile) and mode II (shear), respectively. *q* is the interaction between the two modes. When *q* = 2, Equation (3) becomes the quadratic stress criterion.

**Figure 16:**
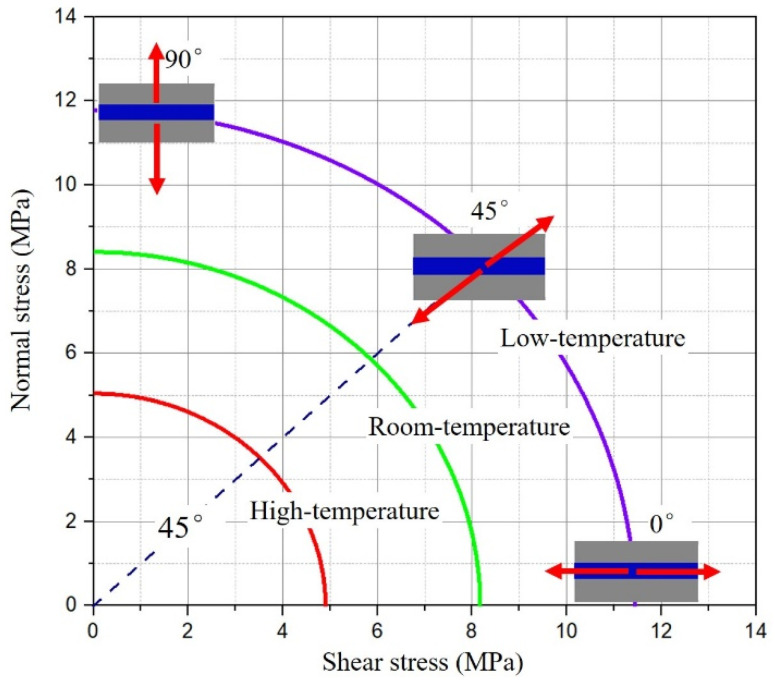
Stress criterion envelope of adhesive joints under whole service temperature.

After the same aging cycle, the normal stress and shear stress in BJ, 45° SJ, and TASJ are dispersed, and the stress criterion envelope is formed in the coordinate system with shear stress as abscissa and normal stress as ordinate. Simultaneously, the stress criterion envelope under the whole service temperature range is made according to the failure stress of adhesive joints at different temperatures, as shown in Figure 16. When the combined state of normal stress and shear stress is outside the envelope, it means that the bonded structure will be destroyed, and any combined stress in the envelope indicates that the bonded structure will not fail.

### 4.2. Establishment of Failure Criterion

**Figure 17:**
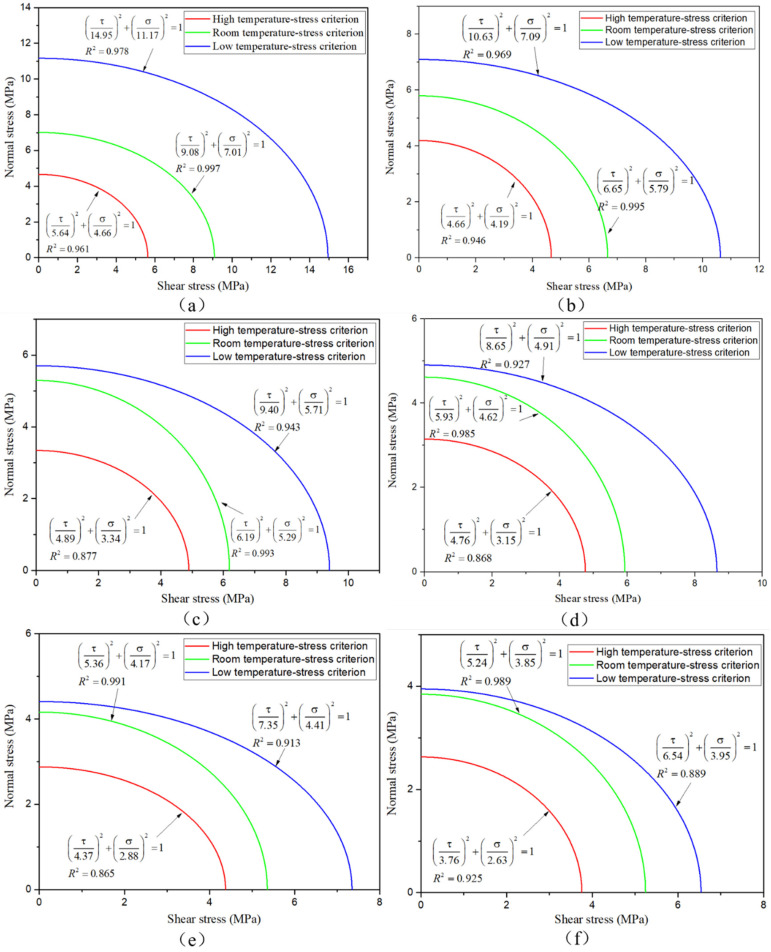
Secondary stress criterion fitting curves: (**a**) unaged; after aging for (**b**) 6, (**c**) 12, (**d**) 18, (**e**) 24, (**f**) 30 days.

To get the failure criterion of joints under different temperatures after hygrothermal aging, the secondary stress criteria in formula (3) are fitted by MATLAB to form the corresponding envelope of the stress criterion [20]. The fitting curves reflect the relationship between normal stress and shear stress, and the fitting accuracy is compared with the *R*^2^ value. The fitting curves, fitting formula, and *R*^2^ value are shown in Figure 17; the secondary stress criterion has an excellent fitting effect.

**Figure 18:**
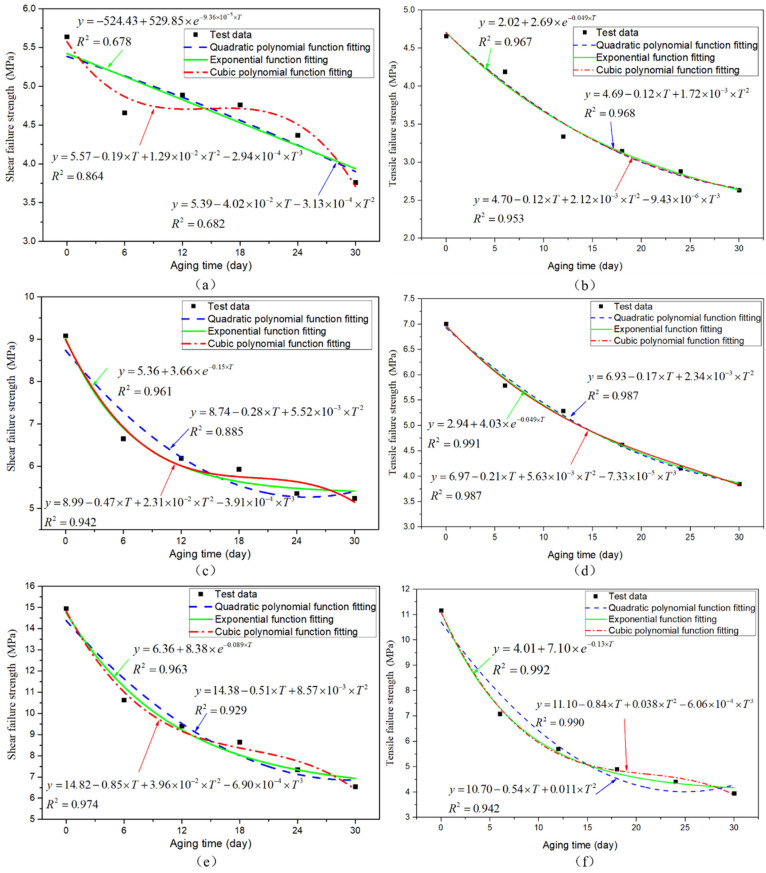
High-temperature test: (**a**) shear failure strength, (**b**) tensile failure strength fitting curve; room-temperature test: (**c**) shear failure strength, (**d**) tensile failure strength fitting curve; low-temperature test: (**e**) shear failure strength, (**f**) tensile failure strength fitting curve.

To evaluate the failure of the adhesive structure after the hygrothermal aging in the application of high-speed trains, the failure criteria of the adhesive joints with different aging periods need to be established by fitting the relationship between the failure criteria and the aging time. Based on the above analysis of failure criteria after 0 (unaged), 6, 12, 18, 24, and 30 days aged, all conform to the secondary stress criterion. It is assumed that the failure criteria of any aging time in 0–30 days are in accordance with the secondary stress criterion. The failure strength values in mode I and mode II are extracted from Figure 17, and their functional relationship with the aging period is fitted. The failure strength and aging time are fitted by selecting the quadratic polynomial, cubic polynomial, and exponential function, as shown in Figure 18. It is found that the fitting accuracy of the cubic polynomial function is relatively best, so it is more appropriate to choose the cubic polynomial function here to establish the failure criterion.

The functional relationship between the failure strength of mode I and mode II with the aging cycle are obtained. The failure criterion function of the adhesive joint at high temperature after hygrothermal aging is shown in Equation (4), the failure criterion function of the joint at room temperature is shown in Equation (5), and the failure criterion function of the joint at low temperature is shown in Equation (6).

(4) $${\left(\frac{\sigma }{4.70-0.12\times T+2.12\times 10^{-3}\times T^{2}-9.43\times 10^{-6}\times T^{3}}\right)}^{2}+{\left(\frac{\tau }{5.57-0.19\times T+1.29\times 10^{-2}\times T^{2}-2.94\times 10^{-4}\times T^{3}}\right)}^{2}=1$$

(5) $${\left(\frac{\sigma }{6.97-0.21\times T+5.63\times 10^{-3}\times T^{2}-7.33\times 10^{-5}\times T^{3}}\right)}^{2}+{\left(\frac{\tau }{8.99-0.47\times T+2.31\times 10^{-2}\times T^{2}-3.91\times 10^{-4}\times T^{3}}\right)}^{2}=1$$

(6) $${\left(\frac{\sigma }{11.10-0.84\times T+0.038\times T^{2}-6.06\times 10^{-4}\times T^{3}}\right)}^{2}+{\left(\frac{\tau }{14.82-0.85\times T+3.96\times 10^{-2}\times T^{2}-6.90\times 10^{-4}\times T^{3}}\right)}^{2}=1$$

where *T* represents the aging duration between 0 and 30 days, and the unit is the day.

**Figure 19:**
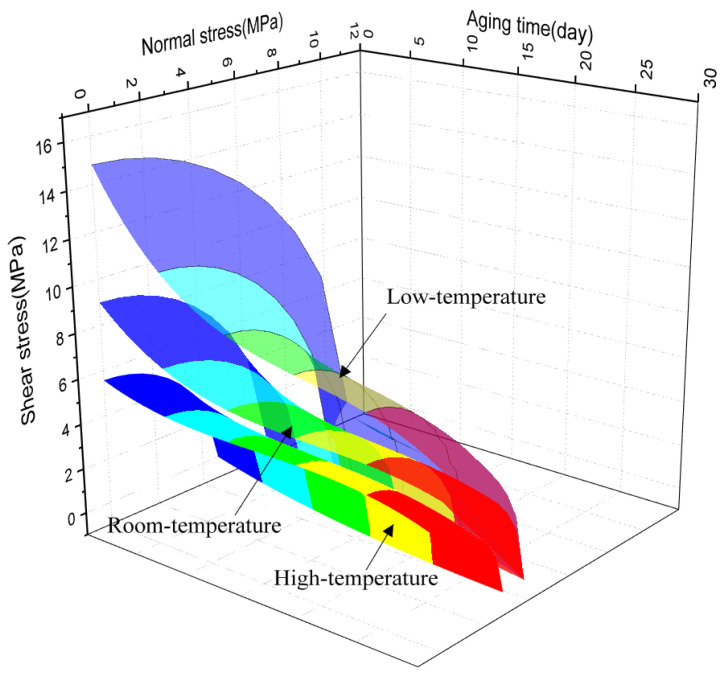
Failure criterion surfaces.

To better show the change of secondary stress criterion with aging time, based on Equations (4)–(6), the three-dimensional surfaces of failure criterion were established by MATLAB software, so that the change of secondary stress criterion with aging cycle could be better explained, as shown in Figure 19. It is found that with the increase of aging time, the envelope of the secondary stress criterion is narrower, which suggests that the strength of the adhesive joints decreases gradually, which indicates that the adhesive structure is more prone to failure. The failure criterion can be used to evaluate the durability of adhesive structures at service temperature.

## 5. Conclusions

In this paper, considering the influence of hygrothermal aging on the adhesive structure, the failure model of the adhesive structure after aging in the service temperature range is established. The mechanical properties and failure modes of joints with different aging levels in the service temperature range are analyzed. The failure mechanism is analyzed through the macro and micro failure section morphology. The failure criteria under the whole service temperature are established, so as to provide reference and basis for the design of the adhesive structure over the whole service temperature range. Based on these results, the following conclusions can be drawn:

(1) The stress state and temperature have significant influence on the failure load of the adhesive joints after hygrothermal aging. The failure load decreases most obviously at low temperature, which is closely related to the failure mode of the joints. With the increase of the normal stress ratio in the joint, the decline of failure load increases gradually.
(2) The failure strength prediction model of joints with different aging levels changing with time at different temperatures was established. The exponential function can obtain very satisfactory fitting accuracy. The fitting effect is better at room temperature and low temperature. The dispersion of joint failure strength is small.
(3) The main failure mode of TASJs is cohesive failure, while BJs are prone to interface failure, and the interface failure is more evident with the decrease of test temperature. The interface failure and the hydrolysis reaction of polyurethane adhesive are the main reasons for the decline of joint strength.
(4) The failure strength of the adhesive joints conforms to the secondary stress failure criterion. The surface equations reflecting the relationship between the quadratic stress criterion, the service temperature, and the aging period were established for the joints.

## Figures and Tables

**Table 1:** Mechanical properties of aluminum alloy.

Young’s Modulus (GPa)	Poisson’s Ratio	Density (kg/m^3^)
71	0.33	2730

**Table 2:** Mechanical properties of adhesive.

Property	Value
Young’s modulus, *E* (MPa)	4.8
Poisson’s ratio	0.48
Tensile failure strength (MPa)	8
Shear failure strength (MPa)	4.5
Fracture elongation (%)	450
Density (kg/m^3^)	1200
Working temperature (°C)	−40~90
Glass transition temperature (°C)	About −45

**Table 3:** Fitting function expressions and fitting accuracy *R*^2^.

Loading Condition	Test Temperature/°C	Function Expression	*R* ^2^
BJ	80	$y=1.82+2.79\times e^{-0.055x}$	0.90
	20	$y=2.61+4.32\times e^{-0.044x}$	0.99
	−40	$y=5.25+6.88\times e^{-0.28x}$	0.96
45° SJ	80	$y=9.97-4.59\times e^{-0.011x}$	0.90
	20	$y=4.42+3.47\times e^{-0.087x}$	0.97
	−40	$y=3.52+7.39\times e^{-0.10x}$	0.99
TASJ	80	$y=3.81+1.52\times e^{-0.17x}$	0.96
	20	$y=5.48+3.54\times e^{-0.18x}$	0.97
	−40	$y=6.18+8.81\times e^{-0.073x}$	0.94

## Data Availability

The data presented in this study are available on request from the corresponding author.
